# Training of practical skills – implementation in the D-A-CH-Region

**DOI:** 10.3205/zma001068

**Published:** 2016-11-15

**Authors:** Martin Lischka

**Affiliations:** 1 Medizinische Universität Wien, Wien, Austria

## Letter to the Editor

Since 1989 the study site in Vienna’s Medical faculty has started its operations step by step inside the New General Hospital. In the editorial on “training of practical skills” the operational start of the “Skills-Lab” is only recognized in 1996. We have therefore checked the developmental history by reference to distinctly diverse documents: design papers, project reports by the study site and university calendars, all of which only exist in print form for this era and are mainly “grey literature”. Equally difficult to find by present-day means are papers presented at congresses and local publications [1], [2].

According to those sources it needs to be recorded that an elective course on training of practical skills was first announced for the winter term 1992/93, the experience of which was presented at the AMEE- congress 1993 and is also reported in a monograph of the Viennese study commission [3], [4].

This investigation has revived the memory that systematic skills training has started in Vienna almost 25 years ago and has brought back a number of questions: what were the supporting factors that had contributed to this early innovation? How has this institution developed from its role as an “early adopter”? What is the current status of scientific documents and publications that are difficult to access? How significant are statements concerning the institution’s seniority anyway? After all various elements for the training of practical skills have been used for a long time, such as obstetrical phantoms, support devices to train auscultation or ophthalmological models (e.g. in Berne, Switzerland). In this respect one would rather have to pose the question why it has taken so long to transform those early approaches into “major projects” – resp. which factors have induced the vigorous development in the “D-A-CH Region” (Germany/Austria/Switzerland) - approximately since the turn of the millennium [5].

## Competing interests

The author declares that he has no competing interest. 
